# A case of brain arteriovenous malformation treated by high-pressure cooker technique assisted with anhydrous alcohol embolization: A case report

**DOI:** 10.1097/MD.0000000000036272

**Published:** 2023-12-15

**Authors:** Cui Zhang, Qingbo Wang, Chenglong Li, Zefu Li

**Affiliations:** a Department of Neurosurgery, Binzhou Medical University Hospital, Shandong, China; b Department of Neurosurgery, Qilu Hospital of Shandong University, Shandong, China.

**Keywords:** brain arteriovenous malformation, curative embolization, endovascular embolization, intent to cure, intracranial hemorrhage

## Abstract

**Rationale:**

Brain arteriovenous malformations (BAVMs) are a relatively rare but very dangerous developmental abnormality of the blood vessels. Intracranial hemorrhage is the most common clinical manifestation of BAVMs, and has a high rate of rebleeding, disability, and mortality, which has a serious impact on patients’ quality of life and working ability. Endovascular intervention was a new technique that emerged in recent years. Anhydrous ethanol embolization has been used with satisfactory results in the treatment of peripheral arteriovenous malformations, but there is a lack of practice in the treatment of BAVMs. We tried to treat BAVMs by embolizing malformed vessels with anhydrous alcohol in order to provide a safe and effective treatment for more patients with BAVMs.

**Patient concerns:**

The patient was admitted to our hospital in the emergency department with “sudden onset of headache for more than 4 hours.” At the time of admission, the patient was clearly conscious, not mentally alert, spoke fluently, and had a normal level of orientations. The direct and indirect responses to light were blunted. The patient’s muscle strength, muscle tone, and sensation of the extremities were normal. National Institute of Health stroke scale score was 1. Head computed tomography at the onset suggested a right occipital hemorrhage and hematoma formation.

**Diagnoses:**

Brain arteriovenous malformations (BAVMs) were suspected based on preoperative imaging findings.

**Interventions:**

After obtaining the consent of the patient and their family members, we performed whole brain angiography to determine the location of the lesion, and then, with the help of high-pressure cooker technology, targeted embolization of interventional BAVM was performed. The high-pressure cooker technology was achieved through spring coils, and the embolic material was anhydrous ethanol.

**Outcomes:**

The surgery was successful, and the patient recovered well without recurrence.

**Lesson:**

The successful performance of this surgery illustrates the feasibility of anhydrous ethanol-targeted ablation for BAVMs.

## 1. Introduction

Brain arteriovenous malformations (BAVMs) are a relatively rare but dangerous vascular developmental anomaly with significant morbidity and mortality.^[1,2]^ When detected, they usually present with spontaneous intracranial hemorrhage (ICH), seizures and focal neurological deficits, such as headaches, and are usually seen in the young population.^[3–7]^ BAVMs initially present as ICH in 36% to 68% of patients.^[8,9]^ Therefore, the management of BAVMs is very important. Treatment modalities for BAVMs include endovascular embolization, surgical resection, and radiological intervention, either as a stand-alone therapy or in combination. Due to the lack of randomized studies comparing treatments, there is still a lack of uniform recommended treatments; with the advent of improved endovascular techniques, sophisticated microcatheter designs, and embolization materials with desirable properties, endovascular embolization of BAVMs has become more common.^[10–13]^ Endovascular embolization of BAVMs has made significant progress few decades since Luessenhop and Spence^[14]^ first described the use of silicone rubber balls to embolize BAVMs in the past few decades; Drugs have also begun to be innovatively applied in large quantities to treatment.^[15,16]^ However, these treatments can have many complications and side effects. In recent years, intravascular anhydrous ethanol embolization has become a new treatment method and has been widely used for extracranial arteriovenous malformations (AVMs).^[17,18]^ The infusion of anhydrous ethanol causes protein denaturation, which directly and extensively damages the endothelial cells, causing them to lose their endocrine function and promoting thrombus formation in the “nest.” This results in permanent vascular occlusion. In one study,^[19]^ the use of anhydrous ethanol embolization for the treatment of BAVMs was found to have a 90% success rate. Nevertheless, there are still some limitations and shortcomings of this treatment method. Anhydrous ethanol is highly toxic to the surrounding normal blood vessels and may cause irreversible damage to the peripheral nerve tissue during the treatment process. In order to minimize the occurrence of complications, and make this technique more acceptable to more patients, the Department of Neurosurgery at our medical center tried to implement the high-pressure cooker technique by embolizing the corresponding arteriovenous communication point of malformed vascular mass with a spring coil to prevent the retrograde reflux of the embolic agent and to help anhydrous ethanol flow forward to the target site. And one case of BAVMs was successfully treated, which is reported as follows.

## 2. Case presentation

**Figure 1.**
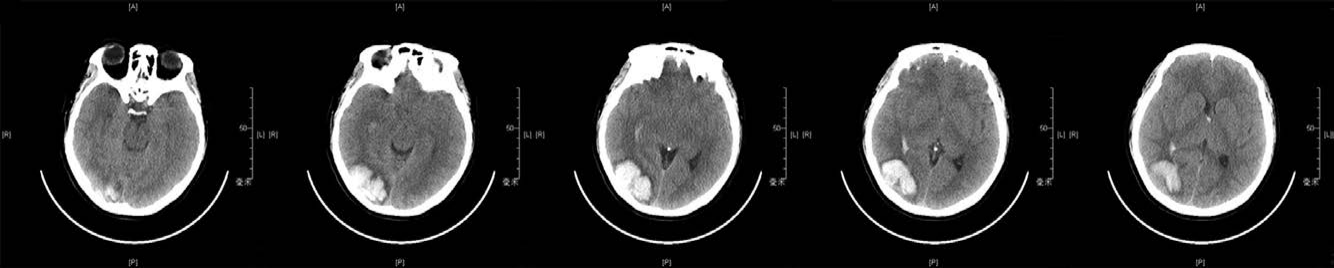
The preoperative CT images of the patient’s brain, showing the patient’s right occipital lobe hemorrhage and hematoma formation. CT = computed tomography.

A 32-year-old female patient was admitted to our hospital in the emergency department with “sudden onset of headache for more than 4 hours.” At the time of admission, the patient was clearly conscious, not mentally alert, spoke fluently, and had a normal level of orientations. She was no cranial deformity. The pupils were about 3 mm in diameter bilaterally, and the direct and indirect responses to light were blunted, with no restriction of eye movements in any direction. The muscle strength of both upper and lower extremities was grade 5, the muscle tone examination was normal, and the sensation of the extremities was normal. National Institute of Health stroke scale score was 1. Cranial computed tomography at the onset suggested a right occipital hemorrhage and hematoma formation (Fig. 1).

**Figure 2.**
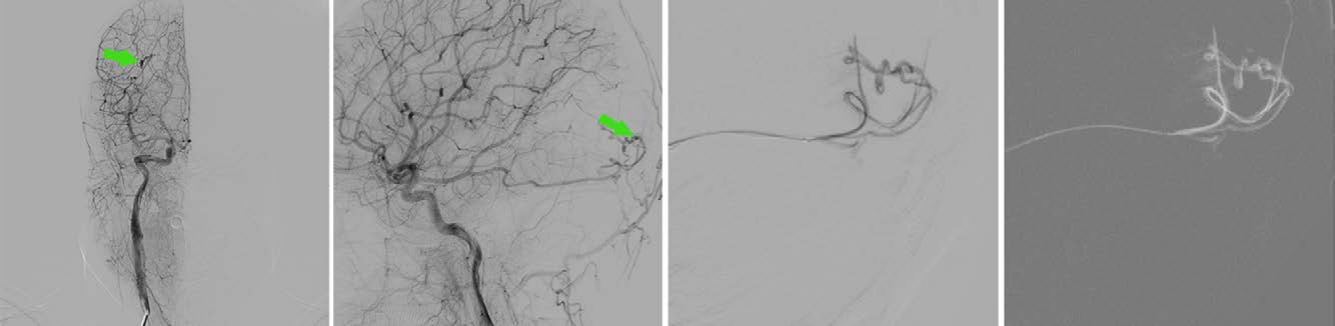
DSA results of the patient before embolization treatment. The green arrows show the malformed vascular mass under contrast medium imaging, which is too small to be about 8 × 15 mm.

**Figure 3.**
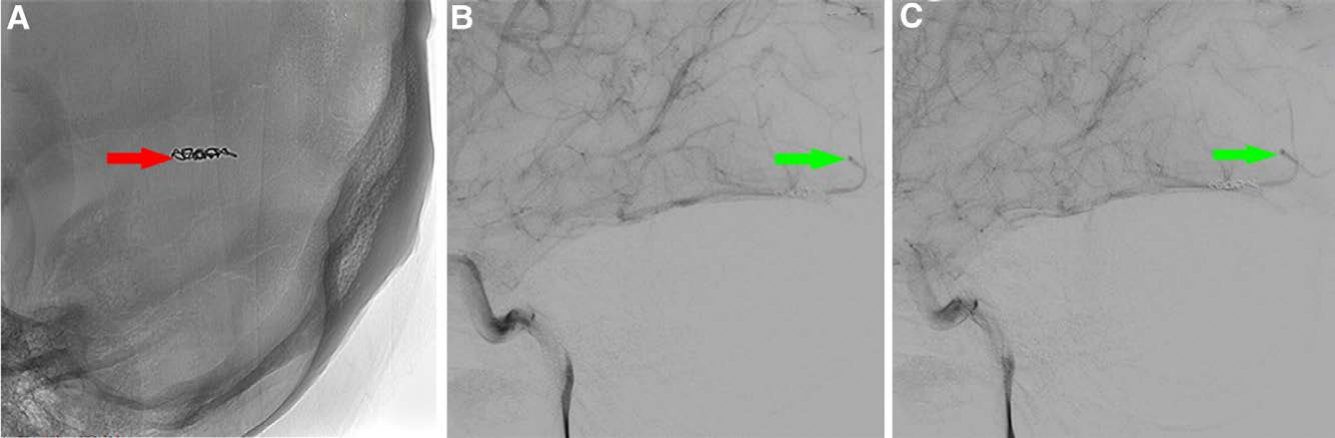
The result of reexamination after alcohol embolization. The red arrow shows the MicroPlex2*8 one coil that the embolism entered (A). The green arrow shows that after alcohol embolization, the original vascular malformation mass is not significantly developed (B and C).

After differential diagnosis and in-hospital multidisciplinary consultation, urgent whole brain angiography was recommended to clarify the cause of cerebral hemorrhage and, based on the angiography, timely endovascular interventional embolization was performed to stop the bleeding at the bleeding point and potential bleeding points. Interventional targeted embolization of BAVMs and whole brain angiography were performed after obtaining the consent of the patient and his family and after approval by the hospital ethics committee: The patient was placed in the supine position, and after satisfactory general anesthesia, routine surgical disinfection of the bilateral inguinal region was performed. The right femoral artery was punctured by Seldinger technique after laying sterile drape sheet, and 8F sheath was placed. 5F contrast catheter (Boston Scientific, USA) was used to perform 3D rotational angiography of the right internal carotid artery, left common carotid artery, right vertebral artery, and left subclavian artery under the cooperation of mudskipper guidewire. The image reconstruction showed that the AVM was seen in the right occipital lobe, which was too small about 8*15 nm. The supplying artery was the distal branch of the right posterior communicating artery, and there was a thick draining vein distal to the malformed mass (Fig. 2) (Video 1, Supplemental Digital Content 1, http://links.lww.com/MD/K826; Video 2, Supplemental Digital Content 2, http://links.lww.com/MD/K827). The guiding catheter was replaced and placed at the opening of the right internal carotid artery. A MicroPlex2*8 spring coil (MicroVention, Inc., USA) was inserted through the Headway17 microcatheter (MicroVention, Inc., USA) and placed in the proximal vessel of the malformed mass (Fig. 3A). Subsequently, 2 ml of contrast medium (Iopamidol 50 ml:18.5 g, CHN) was injected along the SL-10 microcatheter. An aneurysm-like structure was seen on angiography. After 5 minutes of observation, there was no reflux of contrast agent. Finally, 2 mL of 95% ethanol was slowly pushed through the SL-10 microcatheter, and the aberrant vascular mass was not re-developed on review (Fig. 3B and C) (Video 3, Supplemental Digital Content 3, http://links.lww.com/MD/K828), and the procedure went smoothly. Intraoperative bleeding was 5 mL, and the right femoral artery puncture site was closed with a closure device.

**Figure 4:**
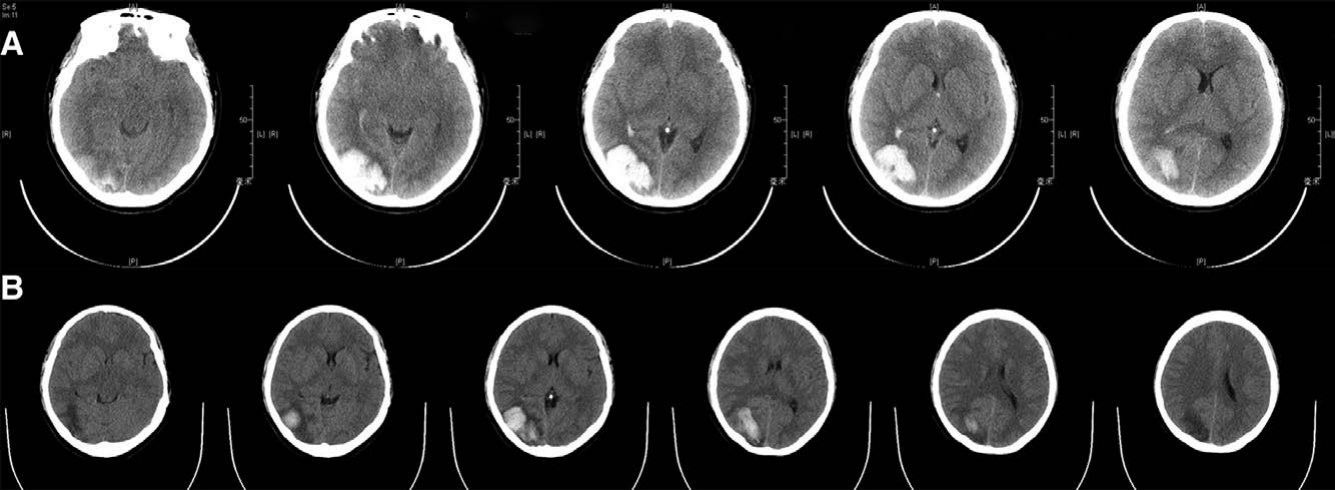
(A) The CT results of the brain within 24 hours after the operation. (B) The CT results of the brain indicating the patient’s treatment status at the time of discharge. CT = computed tomography.

Surgical and follow-up results: Re-examination of cerebral angiography revealed no further development of malformed vascular clusters. With the assistance of pressure cooker technology (which is achieved by MicroPlex2*8 1 coil (MicroVention, Inc., USA)), anhydrous alcohol was successfully used to embolize the deformed vascular masses. After surgery, the patient was transferred to the neurosurgical intensive care unit for blood pressure control for 1 to 3 days, with no significant change in symptoms and the same National Institute of Health stroke scale score as before surgery. Computed tomography findings within 24 hours after surgery showed no significant change from the preoperative period (Fig. 4A), and hematoma resorption was obvious at discharge (Fig. 4B).

## 3. Discussion

BAVMs are of great concern because they are rare but extremely dangerous. The disease is diagnosed in approximately 1.12 to 1.42 per 100,000 people each year.^[20–22]^ The clinical manifestations of BAVMs vary from headache, epilepsy, visual loss, to neurological dysfunction or paralysis. These symptoms have a serious impact on patients’ quality of life and ability to work. More importantly, some patients may develop severe ICH leading to fatal consequences. The annual bleeding rate in BAVMs is approximately 2.10% to 4.12%, with approximately 36% to 68% of patients bleeding at the initial visit. The risk of rebleeding increases after the initial diagnosis of ICH, ranging from 9.65% to 15.42% in the first year, and decreases each year thereafter to 1.70% to 3.67% after 5 years.^[8,9]^ Given the high rebleeding rate of ruptured BAVMs, the need for aggressive treatment of ruptured BAVM has been widely recognized. Therefore, BAVMs diagnosis and treatment is of very necessary importance.

Patients with symptoms and manifestations of BAVMs should be diagnosed and treated early, with the goal of reducing symptoms, preventing ICH, and maximizing quality of life. Current treatment modalities for BAVMs include surgical, interventional, stereotactic radiosurgery, and a combination of these modalities. Interventional therapy is the treatment of choice for BAVMs in most cases.^[23,24]^ Embolization aimed at curing BAVMs has been reported to completely occlude malformed vessels to some extent.^[19]^ The goal of our acute embolization is to rapidly initiate treatment to eliminate rebleeding. Because the specific factors leading to rupture of BAVMs are unknown, acute embolization can be performed regardless of whether the point of rupture is identified^[25]^; complete occlusion rates can exceed 90% when treating highly selective patients with first-stage embolization.^[19]^ Anhydrous ethanol ablation has achieved good results in the treatment of extracranial, head and facial vascular malformations,^[17,18]^ but in intracranial vessels, embolization and acute thrombosis may lead to swelling of brain tissue adjacent to the AVM masses. In addition, ethanol may even exacerbate the swelling due to its chemical irritant effect. Therefore, anhydrous ethanol is not frequently used due to the high risk of complications.^[26]^ However, it is undeniable that anhydrous ethanol is a good sclerosing agent and there are some encouraging results for the efficacy of anhydrous ethanol in the treatment of BAVMs. A 10-year retrospective study conducted at a California medical center found^[27]^ a high success rate of ethanol sclerotherapy techniques and a low complication rate in 18 cases of ruptured BAVMs shaped as blood supply aneurysms and visceral aneurysms. Certainly, Ykes WF et al in their study also showed that edema caused by anhydrous ethanol embolization of AVM masses and adjacent tissues could be controlled by Decadron treatment.^[26]^ The differences in the results of such studies may be related to factors such as the purpose of the procedure and the concentration of alcohol used during the procedure. To ensure the safety of the procedure, the high-pressure cooker technique was applied.

The spring coil embolization of the arteriovenous communication point of the deformed blood vessel mass simulates the use of high-pressure cooker technology to block the blood flow channel between the artery and the deformed vein, increase the pressure of the reflux vein, block the blood flow channel, and make the embolization agent tightly fit between the artery and the deformed vein to achieve the embolization effect. The specific operation method is as follows: we first occluded the arteriovenous communication with a spring coil and injected anhydrous ethanol into the malformed vascular mass through another microcatheter. The ethanol diffuses in the malformed vascular mass, the vascular endothelium contracts, and the stroma is exposed to thrombosis to treat BAVMs. In this treatment, the pressure cooker technique was implemented by 1 MicroPlex2*8 coil (MicroVention, Inc., USA), which prevents the backflow of the embolic agent and helps it to flow forward to the target location. The target vascular malformation mass was successfully ablated using 95% ethanol in combination with the high-pressure cooker technique.

It must be clearly stated that anhydrous alcohol is a dangerous intravascular substance that can be injected. Anhydrous alcohol embolization requires precise injection of alcohol into the target vessel, which may require multiple attempts. This adds to the hassle and risk of the procedure. If the alcohol is mistakenly leaked into surrounding tissue or healthy vessels during injection, tissue damage or vessel blockage may result. Therefore accidental embolization must be avoided by ultra-selective localization, and sufficient proficiency of the operator is also necessary. Furthermore, we have prevented the migration of anhydrous ethanol in non-targeted vessels by means of a pressurized steaming technique and small doses of anhydrous ethanol. It showed promise in complete ablation of BAVMs. Although postoperative angiography showed complete ablation of the target, it remains unknown whether the aneurysmal-like structures are occluded or whether the occluded targets will be recanalized. To avoid such situations, patients need to be followed up for a long period of time and treated promptly when recurrence or recanalization occurs. The long-term complications of ethanol treatment of BAVMs are also of concern. Although no thrombosis-related complications occurred during the patients’ hospitalization (13 days in total), the long-term prognosis of the patients still needs to be investigated over time and follow-up period. In addition, the selection of the appropriate ethanol concentration based on the characteristics of BAVMs and hemodynamics still needs to be further investigated.

## 4. Conclusions

The successful performance of this surgery illustrates the feasibility of anhydrous ethanol-targeted ablation for BAVMs. To take full advantage of the good dispersibility of anhydrous alcohol, the combination of high-pressure cooker technology allows anhydrous ethanol to reach the target area to the maximum extent and effectively prevents its reflux. Likewise, anhydrous ethanol is more readily available and the material cost is lower, making it a valuable option for patients in less economically developed areas or those who cannot afford expensive materials.

## Author contributions

**Conceptualization:** Zefu Li.

**Methodology:** Qingbo Wang, Chenglong Li.

**Resources:** Cui Zhang.

**Supervision:** Zefu Li.

**Writing – original draft:** Cui Zhang.

**Writing – review & editing:** Zefu Li, Cui Zhang.

## Supplementary Material






